# WHIRLY1 Occupancy Affects Histone Lysine Modification and *WRKY53* Transcription in *Arabidopsis* Developmental Manner

**DOI:** 10.3389/fpls.2018.01503

**Published:** 2018-10-19

**Authors:** Dongmei Huang, Wei Lan, Danjing Li, Ban Deng, Wenfang Lin, Yujun Ren, Ying Miao

**Affiliations:** Center for Molecular Cell and Systems Biology, Fujian Provincial Key Laboratory of Haixia Applied Plant Systems Biology, College of Life Sciences, Fujian Agriculture and Forestry University, Fuzhou, China

**Keywords:** ssDNA binding protein, transcription, histone modification, leaf senescence, *Arabidopsis*

## Abstract

Single-stranded DNA-binding proteins (SSBs) are assumed to involve in DNA replication, DNA repairmen, and gene transcription. Here, we provide the direct evidence on the functionality of an *Arabidopsis* SSB, WHIRLY1, by using loss- or gain-of-function lines. We show that WHIRLY1 binding to the promoter of *WRKY53* represses the enrichment of H3K4me3, but enhances the enrichment of H3K9ac at the region contained WHIRLY1-binding sequences and TATA box or the translation start region of *WRKY53*, coincided with a recruitment of RNAPII. *In vitro* ChIP assays confirm that WHIRLY1 inhibits H3K4me3 enrichment at the preinitiation complex formation stage, while promotes H3K9ac enrichment and RNAPII recruitment at the elongation stage, consequently affecting the transcription of *WRKY53*. These results further explore the molecular actions underlying SSB-mediated gene transcription through epigenetic regulation in plant senescence.

## Introduction

Plant senescence is the last stage of plant development. It is a controlled process that plants response to internal factors (age and hormone) and external factors (abiotic stress and biotic stress), in which plants remobilize nutrients from source leaves to developing tissues. In the past decades, molecular components underlying the onset of senescence have been studied, however, the age-dependent mechanisms that control the onset of senescence remains opening.

WRKY is a major transcription factor (TF) family of plants, in which many of them are the central players in gene regulation during leaf senescence. WRKY6 was found to activate several senescence-associated genes during leaf senescence (Robatzek and Somssich, 2002). Mutation and overexpression of WRKY6 retarded and accelerated both developmentally and dark-induced senescence (Robatzek and Somssich, 2002; Zhang et al., 2018). Similarly, overexpression or knockout of *WRKY22* also showed accelerated and delayed senescence phenotype in dark condition (Zhou et al., 2011). WRKY53 and WRKY70 were reported to be positive and negative regulators of senescence, respectively. Loss of WRKY53 delayed the leaf senescence (Miao et al., 2004), while *wrky70* mutant showed aggravated senescent phenotype during development and dark treatment (Ulker et al., 2007). WRKY54 can co-operate with WRKY70 to repress leaf senescence (Besseau et al., 2012). Additionally, WRKY57 function as a repressor in JA-induced leaf senescence (Jiang et al., 2014) and WRKY45 positively regulate age-triggered leaf senescence *via* gibberellin (GA)-mediated signaling pathway (Chen et al., 2017). Mutual regulation exists between WRKYs and WRKYs can be regulated at different levels (Phukan et al., 2016).

WRKY53, a well-known regulator to plant early leaf senescence, is a convergence node tightly regulated by various process (Zentgraf et al., 2010). WRKY53 binds to more than 60 target genes directly (Miao et al., 2004). It has been reported that WRKY53 protein is regulated by MEKK1 kinase, epithiospecifying senescence regulator (Miao and Zentgraf, 2007; Miao et al., 2007), and UPL5 in the protein level (Miao and Zentgraf, 2010). Moreover, the expression of *WRKY53* gene is activated by GATA4 (Zentgraf et al., 2010), activation domain protein (AD protein) (Miao et al., 2008), and REVOLUTA protein (Xie et al., 2014) in the transcriptional level in leaf senescence, while it is repressed by ssDNA binding protein WHIRLY1 (Miao et al., 2013). The histone modification at *WRKY53* locus seemed to associate with SUVH2 histone methylase (Ay et al., 2009). Current research showed WRKY53 can interact with histone deacetylase 9 (HDA9) affecting downstream gene expression in leaf senescence (Chen et al., 2016). The AT-rich motif in WHIRLY1 binding domain of *WRKY53* has been shown to relate to chromatin structure and epigenetic mark modification (Lim et al., 2007). However, up to now, there is no further evidence about the transcriptional regulation of *WRKY53* by single stranded DNA binding WHIRLY1 protein with chromatin modification.

Single-stranded DNA binding proteins are ubiquitous in organisms and essential in recognition and processing of ssDNA during various cellular processes (Dickey et al., 2013). They bind to ssDNA with high sequence specificity or independent of sequence, stabilizing the ssDNA intermediates during several cellular processes, such as DNA replication, recombination, and repair as well as telomere maintenance (Dickey et al., 2013; Ribeiro et al., 2016; Hedglin and Benkovic, 2017; Huang S.H. et al., 2017). SSB proteins is also given a role in the regulation of gene expression (MacDonald et al., 1995; Liu et al., 2000; Kim et al., 2005; Choi et al., 2009; Miao et al., 2013) and regulation of the activity of many other DNA metabolic proteins (Lohman and Ferrari, 1994; Roy et al., 2007; Shereda et al., 2008; Roy et al., 2009). As a single-stranded DNA binding protein, WHIRLY domains are four structural topologies that have been structurally characterized with ssDNA (Dickey et al., 2013). WHIRLY domains are approximately 180-amino-acid-long domains characterized by two roughly parallel four-stranded β sheets with interspersed helical elements (Desveaux et al., 2005; Cappadocia et al., 2013).

As a member of WHIRLY family, WHIRLY1 has been proven to be a plastid nucleoid-associated protein affecting DNA replication (Krupinska et al., 2014b) and have a function in repair of organelle DNA (Etminan et al., 2010; Maréchal and Zou, 2014) and in maintenance of plastid genome stability (Lepage et al., 2013; Zampini et al., 2015). WHIRLY1 has been likewise implicated in telomere maintenance through binding to four copies of the telomere repeat (Yoo et al., 2007). However, WHIRLY1 protein first has been reported to bind to the inverted repeat sequence of the elicitor response element (ERE) on the promoter of *PR-10a* gene in potato, acting as a transcription activator (Desveaux et al., 2000). Then other studies have shown WHIRLY1 together with WHIRLY3 can bind to the AT-rich region of kinesin gene promoter to activate kinesin gene expression in *Arabidopsis* (Xiong et al., 2009). Recently, WHIRLY1 has been reported to bind to the GTCAAT motif of *S40* promoter in barley by Nanoelectrospray Mass Spectroscopy (Krupinska et al., 2014a). In our previous study, we have found that WHIRLY1 can bind to combination motif of GTNNNAAT and AT-rich motif of downstream target genes, such as *WRKY53, WRKY33, SPO11*, and *PR1* by *in vivo* chromatin immunoprecipitation sequencing (ChIP-seq). It has been verified that WHIRLY1 bind to the promoter of *WRKY53* to repress expression of *WRKY53* and *WRKY33* in *Arabidopsis* leaf senescence (Miao et al., 2013; Ren et al., 2017). Nevertheless, how WHIRLY1 as a single-stranded protein mediate the transcription of downstream genes in a fine-tune case remains elusive.

In this study, we first address WHIRLY1 occupancy on *WRKY53* promoter, enrichment of several histone modifications and recruitment of RNAPII at promoter and translation start regions of *WRKY53*, as well as gene expression of *WRKY53* in chronological during leaf aging. Biochemical and genetically evidences demonstrate that WHIRLY1 occupancy at the *WRKY53* promoter affects not only the enrichment of H3K4me3 and H3K9ac but also RNAPII recruitment at WHIRLY1 bind region and translation start region of *WRKY53 in vivo* and *in vitro*, which influences the transcription of *WRKY53* in the development manner. This study provides an implication for exploration of SSB-mediated transcription in epigenetically modification level in plants.

## Materials and Methods

### Plant Materials and Growth Conditions

All the plants used were *Arabidopsis thaliana (L.) Heynold ecotype Columbia 0* background, WHIRLY1 T-DNA insertion line *Salk_023713* (*why1*) were provided by the European Arabidopsis Stock Centre, while WHIRLY1 overexpression mutants (*oepnWHY1* and *oenWHY1*), WHIRLY1 complementary line (*PWHY1*) which harbors its own promoter, and WHIRLY1 CDS plus HA target were constructed at previous research (Miao et al., 2013). Seeds were germinated on wet filter paper after 48 h of vernalization. Then they were transplanted in pots in vermiculite in a climatic chamber with a 13-h light (100 μE/h)/11-h dark photoperiod, 22°C/18°C day–night temperature regime, and 60% relative humidity. Rosette leaves were labeled with colored threads after emergence, as described previously (Hinderhofer and Zentgraf, 2001). 5–8^th^ rosette leaves from *PWHY1* mutants at 6^th^ week were collected for treatment with 3-Deazaneplanocin A (Dzenp) to detect the influence of WHY1 binding affinity and enrichment of H3K4me3, H3K9ac.

### Measurements of Chlorophyll Fluorescence and Chlorophyll Content

Chlorophyll fluorescence of leaf 5 from different developmental plants was measured using a Pocket PEA Chlorophyll Fluorimeter (Hansatech) after 15-minimum dark incubation. The average Fv/Fm value of leaf 5 from at least 12 individual plants was calculated. Chlorophyll concentrations of leaf 5 from 12 different developmental plants were measured by Dualex 4. Three points of each leaf were detected.

### mRNA Preparation and qRT-PCR Analysis

Total RNA from 5^th^ to 8^th^ rosette leaves was isolated according to the manufacturer’s protocol of TransZol UP (TRANSGEN), and was then treated with RNase-free DNase I (EN0521, Thermo Scientific). First-strand cDNA was generated from 1 μg portion of total RNA using RevertAid First-Strand cDNA Synthesis Kit (K1622, Thermo Fisher), following the instruction. PCR was performed to analyze the expression of genes. To determine the relative expression rate, data were normalized to the expression level of wild-type or of 5-week-old plants (which were set to 1) after normalized to the internal control of *GAPC2*. Additionally, three technical replicates of three biological replicates and the determination of a melting curve of the amplified PCR products were carried out.

### *In vivo* ChIP Assay

The ChIP assay was performed with a modified method (Gendrel et al., 2005). About 0.75 g leaves (fifth to eighth leaves of WT and WHIRLY1 mutants during different developmental stages and whole rosettes of darkness-treated plants) were used. The detailed procedure is shown in **Supplementary Material**. The immunoprecipitated DNA was isolated by Universal DNA purification kit (TIANGEN, DP214-03). Purified DNA was analyzed by real-time PCR with specific primers (see **Supplementary Tables**). Relative histone modification levels in WT during leaf development were therefore normalized to the input, while the enrichment in WHIRLY1 mutants was normalized to WT again, referring to ΔΔCt method^1^.

### Recombinant WHY1 Protein Preparation

The recombinant WHY1 protein was expressed in *E. coli* as described by Miao et al. (2013).

### Chromatin Assembly and Transcription Assays

Chromatin was assembled and transcription assays were performed as described by Active & Motif Chromatin Assembly Manuel^2^. pG5ML with promoter of *WRKY53* including mutated WRKY53II [mutant (GTNNNGGT) m1 or mutant (CTNNNNAAAT) m2 WHIRLY1 binding motif] (Miao et al., 2013) or mutated TATA-box or wild-type fragment was used as DNA template. The detail showed in **Supplementary Material**.

### *In vitro* ChIP

*In vitro* ChIP assays were performed as described (Lauberth et al., 2013) (the detail in **Supplementary Material**). The levels of H3K4me3, H3K9ac are given relative to the total H3 levels. *In vitro* ChIP to detect the effect of WHIRLY1 on transcription stage was performed in the presence or absence of 0.01% sarkosyl, which inhibits PIC assembly but does not affect elongation by pre-formed complexes (Cai and Luse, 1987; Hawley and Roeder, 1987) or 800 nM B2 RNA, which inhibits transcription prior to PIC formation (Espinoza et al., 2004).

## Results

### Enrichment of H3K4me2, H3K4me3, and H3K9ac Were Altered at the Promoter Region of *WRKY53* at Senescence Initiation Stage

In previous work, we performed ChIP-Seq and WHIRLY1 was found binding on the promoter of *WRKY53* as a repressor in the developmental manner (Miao et al., 2013). Leaves of 5–8 from plants grown under 80 μmolm^-2^s^-1^ radiation were chosen to analyze the relationship of WHIRLY1 and downstream gene expression at the fine-turn level (**Supplementary Figure S1**). The expression of *RBCS*, a gene encoding the small subunit of ribulose 1,5-bisphosphate carboxylase, was downregulated at the 6^th^ week while the expression of *SAG12* gene encoding a cysteine protease which expressed only in senescent tissues, was drastically induced at 8^th^ week in current case (**Supplementary Figures S1A–C**). Therefore, we inferred that 5^th^ to 7^th^ week was the initiation and early stage of leaf senescence. WHIRLY1 was phosphorylated by calcineurin B-like-interacting protein kinase14 (CIPK14) and accumulated steady in the nucleus after 5W (Ren et al., 2017) (**Supplementary Figure S1E**). Although occupancy of WHIRLY1 on the promoter of *WRKY53* was conversed with the accumulation of nuclear form WHIRLY1, the expression level of *WRKY53* strikingly increased from 5^th^ week to 6^th^ week while the occupancy of WHIRLY1 on the promoter of *WRKY53* declined (**Supplementary Figures S1D,E**). Taken together, these results demonstrate that WHIRLY1 chronologically repressed *WRKY53* transcription and expression *via* binding to its promoter at senescence initiation stage from 5^th^ week to 7^th^ week.

**FIGURE 1:**
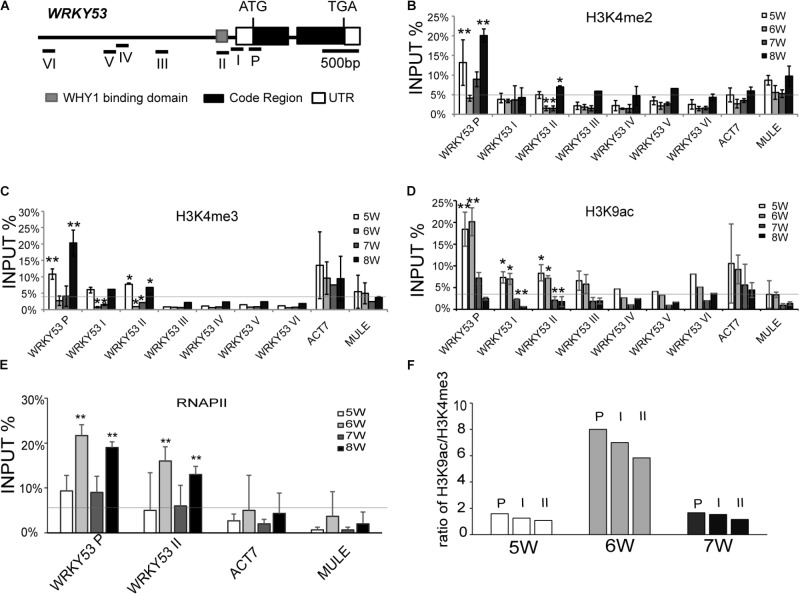
Histone modification and RNAPII occupancy at the promoter regions and translation start region of *WRKY53.* **(A)** Schematic diagram of the genomic structure of the *WRKY53* gene. The lines with number represent qPCR amplicons in different regions of *WRKY53* gene. Gray box, black box, and blank box represent WHIRLY1 binding domain (gray), exon (black), and untranslated regions (blank). **(B–E)** ChIP analyses of changes in H3K4me2 **(B)**, H3K4me3 **(C)**, H3K9ac **(D)** levels, and RNAPII **(E)** occupancy at different regions of *WRKY53* from 5^th^ to 8^th^ week. The antibodies used for ChIP were optimized (**Supplementary Figure S2**). The quantitative PCR was performed with primers (I–VI) around the predicted WHIRLY1 binding sites on the promoter of *WRKY53* and primer (P) covered the 5′ untranslated region and translational start region of *WRKY53* (**Figure 2**) (Ay et al., 2009). *ACT7* (*Actin-related gene 7, AT5G09810*) and *MULE* (*Mutator-like transposable element, AT2G15810*) were analyzed as controls. The relative level was normalized to INPUT DNA. Three biological replicates and three technique replicates were used to analyze. Error bar show the SD (*n* = 3×3). Asterisk indicates significant differences (^∗^*P* < 0.05 and ^∗∗^*P* < 0.01) based on Student’s *t*-test. **(F)** Alteration of ratio of H3K9ac/H3K4me2-3 at different regions of *WRKY53* from 5^th^ to 7^th^ week compared to occupancy of WHIRLY1 and transcripts level of *WRKY53*.

To investigate whether WHIRLY1 binding on the *WRKY53* promoter affect the enrichment of selected histone methylation at senescence initiation stage, chromatin immunoprecipitation experiments were carried out with antibodies against H3K4me2, H3K4me3 (associated with transcriptional active), and H3K27me2 (associated with transcriptional inactive) (**Supplementary Figure S2**) using 5- to 8-week-old wild-type plants. The ChIP assay showed that enrichments of H3K4me2 and H3K4me3 at WRKY53P region which was downstream of WHIRLY1 binding domain at the promoter of *WRKY53* and at WRKY53II region which contained WHIRLY1 binding domain with TATA-box (**Figure 1A**), were at high level in 5-week-old plants, then significantly declined from 5^th^ to 6^th^, and started to increase from 6^th^ to 8^th^ week, and significantly climbed up to 20% high level at 8^th^ week (**Figures 1B,C** and **Supplementary Figure S3**). The enrichment of H3K4me2, H3K4me3 at the residual detected regions of *WRKY53* promoter were at low level compared to that of WRKY53P. H3K27me2 was the lowest at all the detected regions of *WRKY53* gene (**Supplementary Figure S4A**). Although the enrichment of WHIRLY1 at *WRKY53* promoter decreased gradually, still maintained at 40% level at 7^th^ week (**Supplementary Figure S1F**), and *WRKY53* expression level increased rapidly from 5^th^ to 6^th^ week then declined at 7^th^ week (**Supplementary Figure S1D**). Taken together, these results suggest that the occupancy of WHIRLY1 protein at *WRKY53* promoter may be associated with the enrichment of H3K4me2 and H3K4me3 at *WRKY53.* However, WHIRLY1 occupancy did not repress *WRKY53* expression *via* directly correlated with H3K4me2 and H3K4me3.

We also detected the pattern of H3K9ac, H4ac, and RNAPII occupancy at *WRKY53* in wild-type plants during leaf aging. The H3K9ac at promoter and P regions of *WRKY53* maintained high level at 5^th^ and 6^th^ week but decreased sharply from 7^th^ week (**Figure 1D** and **Supplementary Figure S5**). H3K9ac has been reported to positively correlate with the recruitment of RNAPII (Zhang et al., 2017). Expectedly, the recruitment of RNAPII at WRKY53P and WRKY53II regions significantly increased at 6^th^ week, which is consistent with the transcription of *WRKY53* (**Supplementary Figure S6** and **Figure 1E**). The enrichment of H4ac at promoter and P region of *WRKY53* showed a constant low level during leaf senescence in our study (**Supplementary Figure S4B**). Taken together, these results indicate that histone modifications at the promoter and translational start region of *WRKY53* are dynamic from 5^th^ to 8^th^ week and H3K9ac seems more closely related to the transcription of *WRKY53*.

A combinatorial interplay between posttranslational modifications on the same histone was proposed based on the patterns of H3 methylation and acetylation at promoters of specific target genes (Taverna et al., 2007). We calculated the ratio of H3K9ac and H3K4me2-3 at the *WRKY53* promoter during the initiation period of plant senescence from 5^th^ to 7^th^ week. The results showed the ratio pattern of H3K9ac/H3K4me2-3 enrichment was chronologically associated with the occupancy of WHIRLY1 on the *WRKY53* promoter (**Supplementary Figure S1F**) and the transcription level of *WRKY53* **Supplementary Figures S1D,E** and **Figure 1F**) during the initiation period of plant senescence, which suggests that H3K9ac/H3K4me2-3 synergistically control *WRKY53* transcription in plant developmental manner.

### Loss of WHIRLY1 Enhances the H3K4me3 Enrichment at *WRKY53*

**FIGURE 2:**
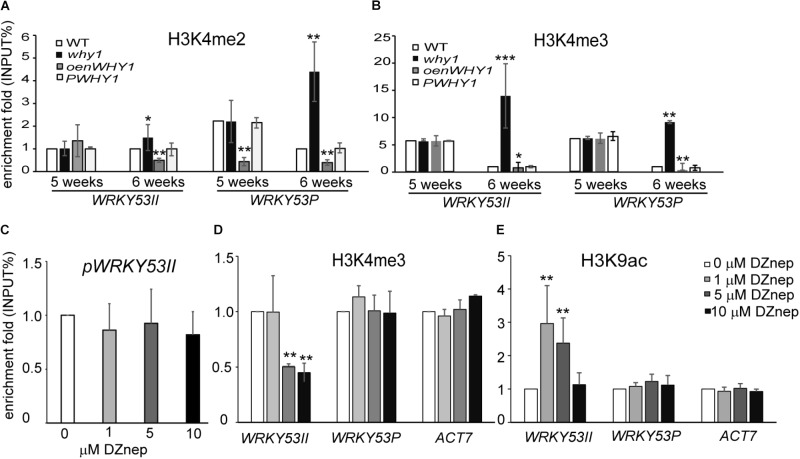
WHIRLY1 regulates the H3K4me3 enrichment at *WRKY53* at senescence initiation stage. **(A,B)** ChIP experiments were performed to assess H3K4me2 **(A)** and H3K4me3 **(B)** levels at WHIRLY1 binding region (WRKY53II) and translation start region (WRKY53P) of *WRKY53* using rosette leaves of *why1, oenWHY1*, wild-type (WT), and *PWHY1* plants. *why1, whirly1*; *oenWHY1, oenWHIRLY1*; and *PWHY1, Pwhirly1:WHIRLY1.* The relative level (input %) was normalized to that of 5-week-old WT. Three biological replicates and three technique replicates were used to analyze. Error bar shows the SD (*n* = 3×3). Asterisk indicate significant differences (^∗^*P* < 0.05, ^∗∗^*P* < 0.01, and ^∗∗∗^*P* < 0.001) based on Student’s *t*-test. **(C–E)** The binding affinity of WHIRLY1 at promoter of *WRKY53* (pWRKY53II) **(C)**, H3K4me3 **(D)**, and H3K9ac **(E)** levels at WRKY53II and WRKY53P regions of *WRKY53* using 6-week-old rosette leaves of *PWHY1* plants after 24 h induction with 1, 5, and 10 μM 3-Deazaneplanocin A, dimethylsulfoxide (Dznep) was used as a control in treatment. *Actin7* was used as an inner control for ChIP–qPCR. ChIP–qPCR were carried out with antibody against HA, H3K4me3, and H3K9ac. Three biological replicates and three technique replicates were used to analyze. Asterisk indicate significant differences (^∗^*P* < 0.05 and ^∗∗^*P* < 0.01) based on Student’s *t*-test.

To further investigate whether the binding of WHIRLY1 affects the enrichment of H3K4me2 and H3K4me3 at promoter and P regions of *WRKY53*, we detected the pattern of H3K4me2, H3K4me3, and RNAPII recruitment at WRKY53II and WRKY53P regions of *WRKY53* in *WHIRLY1* knockout line (*why1*), overexpression nucleus-located WHIRLY1 line (*oenWHY1*), *WHIRLY1* complementary line (*PWHY1*) as well as WT plants (Miao et al., 2013). The ChIP–qPCR results showed that the enrichments of H3K4me2 and H2K4me3 at WRKY53P region of *WRKY53* as well as H3K4me2 at WRKY53II region of *WRKY53* increased significantly at 6^th^ week in *why1* mutant compared to WT plants (**Figure 2A** and **Supplementary Figure S3**), but RNAPII recruitment did not change (**Supplementary Figure S6**). Interestingly, overexpression nuclear isoform WHIRLY1 (Miao et al., 2013) did not have effect on H3K4me2, H3K4me3, and RNAPII recruitment at WRKY53P region, but the enrichment of H3K4me2 at WRKY53II region which contained the WHIRLY1 binding domain decreased (**Figures 2A,B**). The results suggest that loss of WHIRLY1 enhances the H3K4me2 and H3K4me3 enrichment at the 5′ untranslated region and translational start region of *WRKY53* (WRKY53P), while the occupancy of WHIRLY1 seems to inhibit the enrichment of H3K4me2 and H3K4me3 at its binding site on *WRKY53* at senescence initiation stage.

H3K4me3 at promoters and 5′-end regions have been reported to significantly correlate with active gene expression (Santos-Rosa et al., 2002; Zhang et al., 2009; Songa et al., 2014), and the H3K9 acetylation has been supposed to serve as a template for the gain of H3K4me3 marks during leaf senescence (Brusslan et al., 2015). We next investigate whether the enrichment of H3K4me3 affects the WHIRLY1 occupancy and H3K9ac enrichment at *WRKY53*. We treated 6-week-old *PWHY1* plants with different concentrations(0, 1, 5, and 10 μM) of 3-Deazaneplanocin A (DZnep), which is a *S*-adenosyl homocysteine hydrolase inhibitor and has been reported to affect H3K4me3 enrichment at the promoters of 3% genes in *zebrafish* (Ostrup et al., 2014). The results showed that DZnep deduce the enrichment of H3K4me3 at WRKY53II region of *WRKY53* in a dose-dependent manner (**Figure 2D**). However, DZnep had no effect on the occupancy of WHIRLY1 at *WRKY53* promoter (**Figure 2C**). Interestingly, DZnep treatment resulted in increase of H3K9ac at WRKY53II region of *WRKY53* (**Figure 2E**). Therefore, H3K4me3 marks at *WRKY53* promoter did not directly affect the occupancy of WHIRLY1 at *WRKY53* promoter, but H3K4me3 marks correlate with H3K9ac.

### WHIRLY1 Enhances H3K9 Deacetylation and Represses RNAPII Recruitment at *WRKY53* During Early Leaf Senescence

**FIGURE 3:**
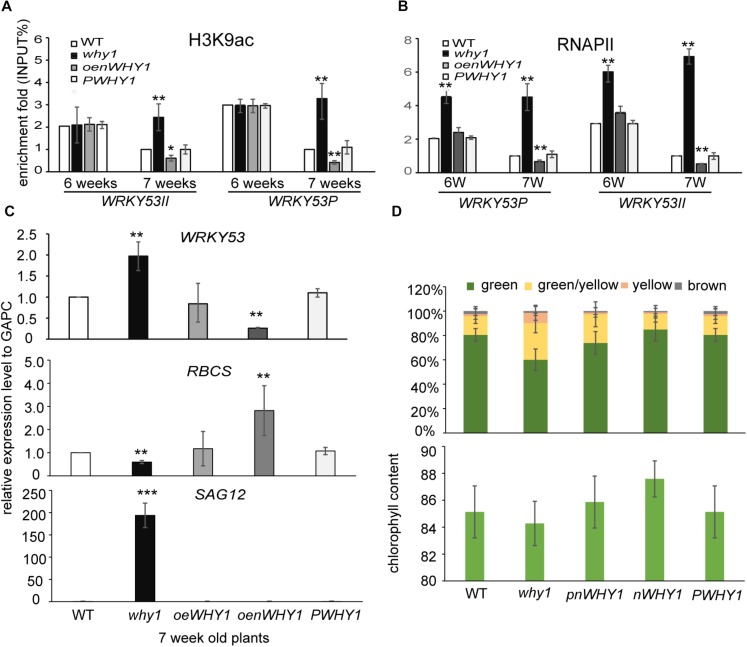
WHIRLY1 enhances H3K9 deacetylation and represses RNAPII recruitment at *WRKY53* at leaf early senescence stage. **(A,B)** ChIP experiments were performed to assess H3K9ac **(A)** and RNAPII occupancy **(B)** at WHIRLY1 binding region (WRKY53II) and translation start region (WRKY53P) of *WRKY53* using rosette leaves of *why1, oenWHY1*, wild-type (WT), and *PWHY1* plants. The relative level (input %) was normalized to that of 5-week-old WT. Three biological replicates and three technique replicates were used to analyze. Error bar shows the SD (*n* = 3×3). Asterisk indicate significant differences (^∗^*P* < 0.05 and ^∗∗^*P* < 0.01) based on Student’s *t*-test. **(C)** Expression of senescence-associated genes in 7-week-old *why1, oenWHY1*, WT, and *PWHY1* plants. The transcript level in each case was normalized to that of *GAPC2* as a reference gene and the expression level of WT was set as 1. Three biological replicates and three technique replicates were used to analyze. Asterisk indicate significant differences (^∗^*P* < 0.05, ^∗∗^*P* < 0.01, and ^∗∗∗^*P* < 0.001) based on Student’s *t*-test. **(D)** Senescent leaf fraction and chlorophyll content in 7-week-old *why1, oenWHY1*, WT, and *PWHY1* plants. Mean and SD of at least 12 independent measurements are shown. Error bars represent SE.

To determine whether WHIRLY1 occupancy affects H3K9ac level and RNAPII recruitment at *WRKY53*, we performed ChIP experiment in *why1, oenWHY1, PWHY1*, and WT plants with anti-H3K9ac and anti-RNAPII antibody (**Supplementary Figure S2**). As showed in **Figure 3** and **Supplementary Figure S5**, the loss of WHIRLY1 led to an increase in H3K9ac at WRKY53II region of *WRKY53* at 6-week-old and 7-week-old wild-type plants, and enhanced the H3K9ac enrichment at WRKY53P region of *WRKY53* at 7-week-old wild-type plants. H3K9ac enrichment at WRKY53P region of *WRKY53* was inhibited at 6-week-old and 7-week-old *oenWHY1* mutant, while H3K9ac enrichment at WRKY53II region of *WRKY53* was only inhibited at 7-week-old *oenWHY1* mutant (**Figure 3A**). Interestingly, significant enrichment of RNAPII at WRKY53II and WRKY53P regions of *WRKY53* were also detected in the 7-week-old *why1* mutants. Overexpression of *WHIRLY1* reduced the RNAPII recruitment at WRKY53II and WRKY53P regions of *WRKY53* at 7-week-old *PWHY1* (**Figure 3B**). As shown in the **Figures 3C,D**, higher transcript level of *WRKY53* and *SAG12* in the *why1* mutant and lower transcript level of *WRKY53* and *SAG12* in the *oenWHY1* were detected, showing high proportion of yellow leaves and less chlorophyll content in the *why*, reversely, less proportion of yellow leaves and high chlorophyll content in the *oenWHY1* line. These results demonstrated that WHIRLY1 binding on *WRKY53* accelerated the deacetylation of H3K9ac and repressed RNAPII recruitment at promoter and translational start region of *WRKY53* to determine *WRKY53* transcript level and senescence-related parameter in a developmental manner.

### WHIRLY1 Protein Occupied on the *WRKY53* Promoter Impacts H3K4me3, H3K9ac, and *WRKY53* Transcription Initiation *in vitro* at the Preinitiation Conformation Stage

**FIGURE 4:**
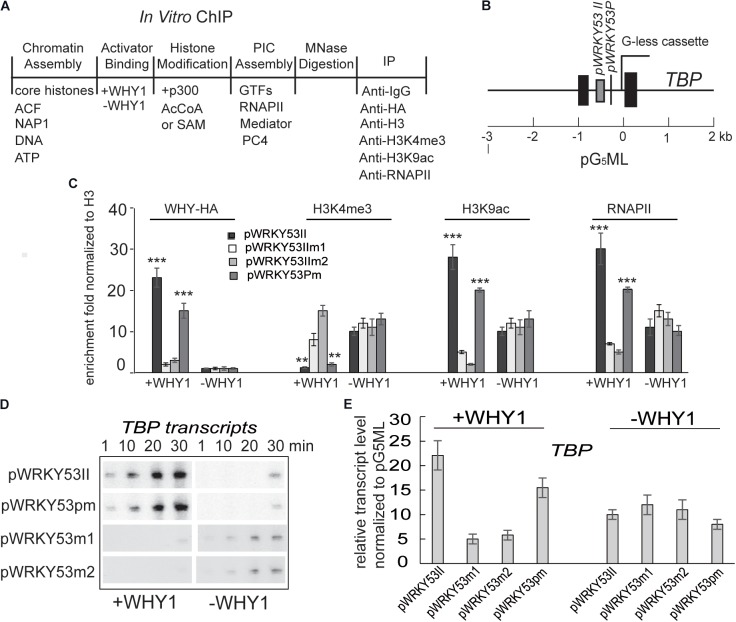
WHIRLY1 impacts H3K4me3, H3K9ac levels on *WRKY53* promoter *in vitro.* **(A)** Schematic of the *in vitro* ChIP assays. **(B)** Schematic of the pG5ML template indicating the amplicons used for qRT-PCR. **(C)** *In vitro* ChIP was performed with assembled chromatin. Chromatin was incubated with or without recombinant WHIRLY1 protein expressed in *E. coli* (Miao et al., 2013) acetylated by p300 or methylated by *S*-adenosyl-l-methionine (SAM), digested with MNase, and immunoprecipitated with antibodies against WHIRLY1, H3, H3K4me3 or H3K9ac, or RNAPII **(A)**. The ChIP-quantitative PCR was carried out using primer containing WHIRLY1 binding sites (WRKY53II) and TATA box. H3K4me3 and H3K9ac levels were relative to H3 levels. Three biological replicates and three technique replicates were used to analyze. Asterisk indicate significant differences (^∗∗^*P* < 0.01 and ^∗∗∗^*P* < 0.001) based on Student’s *t*-test. Error bars represent SE. **(D)** The report gene transcription by run-on assay. **(E)** The report gene transcription level by qRT-PCR. Three biological replicates and three technique replicates were used to analyze. Error bars represent SE.

To confirm occupancy of WHIRLY1 protein at the *WRKY53* promoter directly impacts H3K4 methylation, H3K9ac and *WRKY53* transcription initiation, we recruited a cell-free transcription system (**Figure 4A**) (An and Roeder, 2004). Chromatin was assembled by recombinant factors using Hela core histone and pG_5_ML template with the promoter of *WRKY53* including mutated WRKY53II or mutated TATA-box or wild-type fragment (**Figure 4B**). Micrococcal nuclease digestion showed that all templates are chromatinized equivalently (**Supplementary Figure S7**). *In vitro* ChIP was performed with assembled chromatin. Chromatin was incubated with or without recombinant WHIRLY1 protein expressed in *E. coli* (Miao et al., 2013) and immunoprecipitated with antibodies against WHIRLY1, H3, H3K4me3, or H3K9ac or RNAPII. The ChIP-quantitative PCR was carried out using primer containing WHIRLY1 binding sites (WRKY53II) and TATA box. The enrichment of WHIRLY1 on chromatin templates with wild-type WRKY53II and mutant TATA was obviously higher than that on chromatin templates with mutant WRKY53II (WRKY53IIm1 and WRKY53IIm2) (**Figure 4C**) (Miao et al., 2013). Interestingly, on chromatin templates with wild-type WRKY53II and mutant TATA, in the presence of WHIRLY1, H3K4me3 enrichment was inhibited while H3K9ac enrichment and RNAPII recruitment were enhanced (**Figure 4C**). Further the transcript run-on assay and quantitative RT-PCR were used to detect the transcript elongation and accumulation of *TBP* report genes. Surprisingly, the results showed that report gene *TBP* transcript elongation and accumulation were increased with the time course in the presence of WHIRLY1, while *TBP* transcription did not process when pWRKY53II were mutated or in the deficiency of WHIRLY1 (**Figures 4D,E**). Together, these results indicate that WHIRLY1 binding on WRKY53II *in vitro* represses the enrichment of H3K4me3 and enhance the enrichment of H3K9ac and RNAPII recruitment, and activate the *TBP* transcription.

**FIGURE 5:**
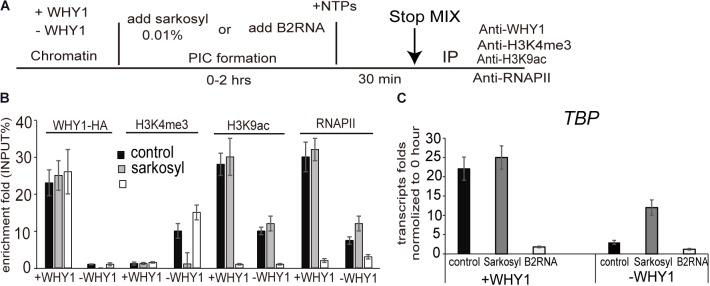
WHIRLY1 affects H3K4me3 and H3K9ac at the preinitiation conformation stage. **(A)** Schematic of the *in vitro* transcription assay. **(B)** ChIP experiments were performed on chromatin assembled *in vitro* after transcription in presence or absence of 0.01% sarkosyl or 800 nM B2RNA with indicated antibodies. H3K4me3 and H3K9ac levels were relative to H3 levels. Three biological replicates and three technique replicates were used to analyze. Error bars represent SE. **(C)** The report gene transcription level by qRT-PCR. Three biological replicates and three technique replicates were used to analyze. Error bars represent SE.

H3K4me3 has been reported to involve in the preinitiation complex (PIC) assembly during transcription (Lauberth et al., 2013; Songa et al., 2014). We wondered WHIRLY1 occupancy on the *WRKY53* promoter impact H3K4me3 before or after PIC formation, therefore, 0.01% sarkosyl, which inhibits PIC assembly but does not affect elongation by pre-formed complexes (Cai and Luse, 1987; Hawley and Roeder, 1987), or B2 RNA, which binds to RNA polymerase II and inhibits transcription before PIC formation (Espinoza et al., 2004), was used to treat assembled chromatin in presence or absence of WHIRLY1 (**Figure 5A**). ChIP–qPCR results showed that neither sarkosyl nor B2 RNA affected the enrichment of WHIRLY1 at the *WRKY53* promoter. However, sarkosyl treatment decreased the enrichment of H3K4me3 at *WRKY53* promoter while B2 RNA treatment inhibited enrichment of H3K9ac and RNAPII recruitment at *WRKY53* promoter (**Figure 5B**). Occupancy of WHIRLY1 on *WRKY53* promoter also inhibited the enrichment of H3K4me3 in the presence of B2 RNA and enhanced the enrichment of H3K9ac and RNAPII recruitment in the presence of sarkosyl (**Figure 5B**). The accumulation of *TBP* transcript was shown in the presence of sarkosyl (**Figure 5C**). These results suggest that WHIRLY1 binding to the *WRKY53* promoter represses the enrichment of H3K4me3 at the preinitiation stage of PIC formation, while promotes the enrichment of H3K9ac and RNAPII recruitment during the elongation by pre-formed complexes, consequently promotes the *TBP* transcription

### WHIRLY1 Regulates the HDACs Expression

**FIGURE 6:**
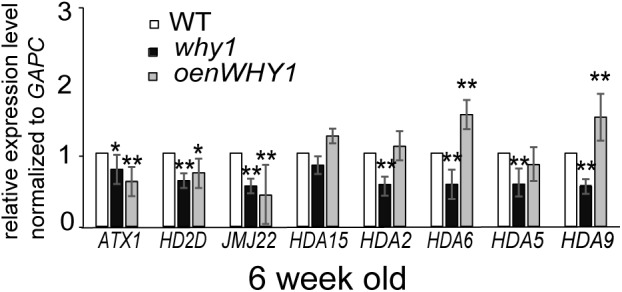
WHIRLY1 affects the HDAs gene expression. The expression of histone modification-related genes in 6-week-old *why1, oenWHY1*, and wild-type (WT) plants by qRT-PCR. The transcript level in each case was normalized to that of *GAPC2* as a reference gene, and the expression level at WT was set as 1. Three biological replicates and three technique replicates were used to analyze. Error bars represent SE. Asterisk indicate significant differences (^∗^*P* < 0.05 and ^∗∗^*P* < 0.01) based on Student’s *t*-test.

To investigate whether WHIRLY1 regulates H3K4me3, H3K9ac, and RNAPII recruitment at the *WRKY53* promoter in transcriptional level, differentially expression genes related to histone modifications were picked from the dataset of RNA-seq between *whirly1* and wild-type plants (Lin et al., 2018, unpublished data; **Supplementary Figure S8**). *SWI3D, HD2D, JMJ22, NFA2*, and *HTA4* were selected to be further analyzed in 6-week-old *why1, oenWHY1* mutants as well as wild-type plants by qRT-PCR. Surprisingly, our qPCR results showed the expression of *HD2D* and *JMJ22* in both *why1* mutants and *oenWHY1* mutants was lower than that of WT (**Figure 6**), which contradicts with the previous RNA-seq data. Overexpressing or knocking out *WHIRLY1* has no effect on gene expression of *SWI3D, NFA2*, and *HTA4*. Moreover, the expression levels of histone methyltransferase *ATX1* (*Arabidopsis Trithorax-like protein 1*), *ATX2, SUVH2*, and histone deacetylases *HDA15, HDA6, HDA2, HDA5*, and *HDA9* were also detected by qPCR. ATX1 and ATX2 play roles for trimethylating and dimethylating K4 of histone H3, respectively (Saleh et al., 2008), and SUVH2 involved in regulating histone methylation marks at *WRKY53* (Ay et al., 2009). The expression of *ATX1* was slightly increased in the *why1* and decreased in the *oenWHY1* mutants, while *ATX2* and *SUVH2* showed no difference in expression between WHIRLY1 mutants and wild-type plants (**Figure 6**). Hence, WHIRLY1 is likely mediated histone methylation by regulating expression of histone methyltransferases ATX1. *HDA2, HDA5, HDA6, HDA9*, and *HDA15* which has been shown highly expression in leaves based on *Arabidopsis* eFP database^3^ were also selected for qPCR. Interestingly, the loss of WHIRLY1 decreased the transcription level of *HDA2, HDA5, HDA6*, and *HDA9*, while overexpressing WHIRLY1 only increased *HDA6* and *HDA9* expression. These results suggest that WHIRLY1 eFP database might involve in regulating gene expression of histone deacetylases to remove the H3K9ac marks at the promoter of *WRKY53*.

## Discussion

WHIRLY1 was reported to be a repressor binding on the GNNNAAATT, plus an AT-rich telomeric repeat-like sequence in the *WRKY53* promoter (Miao et al., 2013). In this study, we have found that enrichment of H3K4me3 and H3K9ac at promoter region contained WHIRLY1 binding domain and TATA box and translation start region of *WRKY53*, and recruitment of RNAPII, as well as transcription of *WRKY53* are coordinated by WHRLY1 protein in a developmental manner. It demonstrates that the occupancy of WHIRLY1 represses the enrichment of H3K4me3 before senescence initiation and enhances the enrichment of H3K9ac and the recruitment of RNAPII at senescence initiation stage, and high ratio of H3K9ac/H3K4me2-3 determines the transcription level of *WRKY53* and leaf senescence initiation. It illuminates that WHIRLY1 works as repressor of *WRKY53* transcription associated with H3K4me2-3/H3K9ac balance in the developmental manner.

### WHIRLY1 Spatio-Temporally Affects H3K4 Methylation and H3K9 Acetylation at *WRKY53* During Leaf Aging

H3K4me2/3 is high at promoter or around the transcription start site (TSS) regions of active or poised genes in animals, while H3K4me2/3 is enriched at the proximal promoter and TSS site of genes in plants (Xiao et al., 2016). Both in animals and plants, H3K4me2/3 as well as H3K9ac which is also enriched at 5′-end and ATG site of genes were reported to correlate with active gene expression (Zhou et al., 2010; Songa et al., 2014). In *Arabidopsis*, H3K4me2/3 mark at 5′untranslated region and translational start region (from -155 bp to +27 bp) as well as gene body (from +367 bp to +549 bp) of *WRKY53* locus increased in senescent leaves which consisted with the expression of *WRKY53* (Ay et al., 2009). However, currently, genome-wide data of ChIP-Seq and RNA-seq showing a constant high level of H3K4me3 and H3K9ac marks in *WRKY53* gene did not directly relate to *WRKY53* expression (Brusslan et al., 2015). *WRKY53* is one example of the many genes that are marked before significant up-regulation of mRNA levels during plant aging. It was explained that this inconsistencies between H3K4me3 marks and gene expression might be down-regulated by WHIRLY1 (Miao et al., 2013; Brusslan et al., 2015), or which may explain the coincidence of low transcript levels and high levels of H3K4me3 marks, as well as the examples of posttranscriptional regulation mediated by small RNAs have been identified during leaf senescence (Kim et al., 2009; Thatcher et al., 2015; Swida-Barteczka et al., 2018). In this study, we clearly showed only some specific regions of *WRKY53* locus showed significantly change in H3K4me2-3 and H3K9ac at specific time point (**Figure 1**). The observation that the level of both H3K4me2/3 and H3K9ac at selected regions (WRKY53II and WRKY53P) of *WRKY53* from 5^th^ to 7^th^ week correlated with the enrichment of WHIRLY1 protein binding on *WRKY53* promoter. It indicates that WHIRLY1 spatio-temporally affect H3K4 methylation and H3K9 acetylation at *WRKY53* during leaf aging. *In vitro* assay further confirms WHIRLY1 binding on specified motifs (GNNNAAATT) of *WRKY53* promoter repressed enrichment of H3K4me3 and potentiated enrichment of H3K9ac, RNAPII recruitment, and *TBP* report gene transcription (**Figure 4**), implicating that WHIRLY1 involves in modification of local chromatin states.

### WHIRLY1 Affects Coordinately the Enrichment of H3K4me3 at the PIC Formation Stage and H3K9ac at the Elongation Stage

Both H3K4me3 and H3K9ac are closely associated with active genes and play an important role in transcription. In human cells, H3K9ac potentiates the interaction of H3K4me3 and basal TF TFIID (Vermeulen et al., 2007). There are numerous studies demonstrated a strong relationship between TF occupancy and chromatin features (Liu et al., 2016). In this study, WHIRLY1 binding on *WRKY53* promoter affected H3K4me3 and H3K9ac *in vivo* and *in vitro*. We utilized two transcription inhibitor sarkosyl and B2 RNA to address this question (Lauberth et al., 2013). Sarkosyl inhibits PIC assembly but does not affect elongation by pre-formed complexes (Cai and Luse, 1987; Hawley and Roeder, 1987), while B2 RNA binds to RNA polymerase II and inhibits transcription before PIC formation (Espinoza et al., 2004). Results of *in vitro* cell-free experiments indicated that WHIRLY1 occupied on *WRKY53* promoter inhibit enrichment of H3K4me3 at the preinitiation stage of PIC formation, in contrary, WHIRLY1 enhances the enrichment of H3K9ac and RNAPII recruitment during the elongation by pre-formed complexes (**Figure 5**).

Compass-like complex is known to facilitate PIC assembly and generate H3K4me3 (Songa et al., 2014), our results showed WHIRLY1 which did not interact with core components of compass-like complex and H3K4 methyltransferases ATX1 then affected the enrichment of H3K4me3. WHIRLY1 binding motif at *WRKY53* promoter was upstream and close to TATA box, in addition, the structure analysis of transcription initiation by RNA polymerase II showed PIC form around TATA box (Grunberg and Hahn, 2013), WHIRLY1 binding on promoter of *WRKY53* may block the site for TATA-box binding protein and repress H3K4me3 enrichment by inhibiting the PIC assembly.

A possible combinatorial interplay between posttranslational modifications on the same histone was proposed based on the patterns of H3 methylation and acetylation at promoters of specific target genes (Taverna et al., 2007). Interestingly, our study suggested a relationship between the enrichment of H3K4me3 and H3K9ac at *WRKY53* promoter region (WRKY53II). Enrichment of H3K4me3 at WRKY53II was inhibited by histone methylation inhibitor DZnep, while the enrichment of H3K9ac was improved by Dznep treatment, in turn, the enrichment of H3K9ac at WRKY53II region decreased in *oehada15* mutant plants while H3K4me3 level at WRKY53II region increased in them. Many protein complexes and epigenetic modifications in the local chromatin environment are necessary for the progress of RNAPII-mediated transcription (Lauberth et al., 2013; Stasevich et al., 2014; Gates et al., 2017). H3K4me3 and H3K9ac were reported to involve in transcription initiation and elongation (Santos-Rosa et al., 2002; Ding et al., 2012; Gates et al., 2017; Zhang et al., 2017) and the interplay between H3K4me3 and H3K9ac at gene promoter was mediated by several factors such as HATs general control nonderepressible 5 (GCN5) (Foulds et al., 2013). WHIRLY1 was involved in the transcription of *WRKY53* by interplay between H3K4me3 and H3K9ac.

### WHIRLY1 Coordinates With HDACs to Modulate the Transcription of *WRKY53*

**FIGURE 7:**
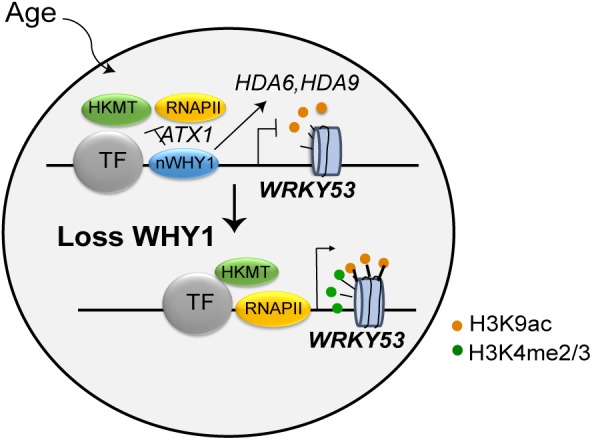
Working model of WHIRLY1 regulates *WRKY53* transcription. WHIRLY1 binding on *WRKY53* promoter represses enrichment of H3K4 methylation by ATX1 inhibiting the PIC formation in leaf senescence initiation and modulates the enrichment of H3K9ac by association with HDACs and high-level transcription at early senescence stage. In absence of WHIRLY1, other transcription activators recruit histone lysine methyltransferase and HDACs is dissociated from *WRKY53* resulting in elevated H3K4me3, H3K9ac levels, and higher occupancy of RNAPII in *WRKY53* loci, thus increasing the transcription of *WRKY53*. TF, transcription factor; HDACs, histone deacetylases; RNAPII, RNA polymerase II; HKMT, histone lysine methyltransferase; nWHY1, nuclear WHY1 protein.

WRKY53 is a well-known transcription factor, plays remarkable role in leaf senescence and plant senescence initiation (Hinderhofer and Zentgraf, 2001; Miao et al., 2004, 2007). It regulated more than 60 senescence-related gene expressions and their related signaling networks, showing delaying senescence phenotype in the *wrky53* line, early senescence phenotype in the *oeWRKY53* lines (Miao et al., 2004, 2007). The expression of *WRKY53* is tightly controlled by multiple layers of regulation, including at the level of chromatin and transcription, as well as by ubiquitination and phosphorylation regulation. (Ay et al., 2009; Miao and Zentgraf, 2010; Zentgraf et al., 2010; Xie et al., 2014; Chen et al., 2016). In this study, WHIRLY1 had been found to bind on the promoter of *WRKY53* and regulate the enrichment of H3K4me3 and H3K9ac in a developmental manner. It may seem paradoxical that WHIRLY1 itself binding on *WRKY53* promoter enhances the enrichment of H3K9ac at leaf senescence initiation while it affects HDACs gene expression to promote the deacetylation of H3K9 at early senescence stage (**Figure 7**). Occupancy of WHIRLY1 on *WRKY53* promoter mainly occurs as *WRKY53* is being shutdown and decreases followed by aging. However, *in vitro* ChIP assay WHIRLY1 increased H3K9ac enrichment and RNAPII recruitment and upregulate downstream target gene transcription. It is a possibility that WHIRLY1 protein not only acts as a block of *WRKY53* transcription before senescence initiation, inhibits activated transcription, but also acts as an activator of other downstream genes such as *PR10a* in potato or *S40* in barley (Desveaux et al., 2000; Krupinska et al., 2014a), which might be coordinated with chromatin remodeling factor BRM or HDA6, HDA19 (Efroni et al., 2013; Chen et al., 2016). Additionally, the occupancy of WHIRLY1 may affect the accessibility of DNA by adjusting themselves to be more complicated complex under specific signals (Cappadocia et al., 2012). This may be the reason why the expression of *HD2D* and *JMJ* decreased in both *why1* mutants and *oenWHY1* mutants.

Conclusively, we propose a model that WHIRLY1 binding on *WRKY53* promoter represses enrichment of H3K4 methylation by ATX1 inhibits the PIC formation in leaf senescence initiation and modulates the enrichment of H3K9ac by association with HDAs and high-level transcription at early senescence stage (**Figure 7**). Further, what signals or factors promoted and controlled the action of WHIRLY1 on *WRKY53* promoter during leaf senescence are still speculated. The accumulation of WHIRLY1 in nucleus is mediated by CIPK14 kinase which was promoted by light conditions, sugar, cytokinin, and calcium–calmodulin signal (Gan and Amasino, 1995; Lee et al., 2005; Akaboshi et al., 2008; Qin et al., 2010; Yan et al., 2014; Ren et al., 2017). Indeed, WHIRLY1 involved in response to different light conditions (Huang D. et al., 2017; Kucharewicz et al., 2017), further revealing that their relationships might provide insights into the upstream signals of WHIRLY1 function on SSB binding on target genes.

## Author Contributions

YM designed the study and performed *in vitro* ChIP assay. DH and BD performed ChIP–qPCR, Western blots, and phenotyping. WL performed qRT-PCR. DL crossed double mutant and screen them, phenotyping. WfL and YR for qRT-PCR and RNA-seq data analyses. DH and YM analyzed the data and wrote the paper.

## Conflict of Interest Statement

The authors declare that the research was conducted in the absence of any commercial or financial relationships that could be construed as a potential conflict of interest.

## Footnotes

1 https://www.qiagen.com/us/resources/resourcedetail?id=34e05db7-689c-4abc-bde6-6488d097394f&lang=en

2 www.activemotif.com

3 http://bbc.botany.utoronto.ca/
