# Supplementary material for: WHIRLY1 Occupancy Affects Histone Lysine Modification and WRKY53 Transcription in Arabidopsis Developmental Manner
Source: Front Plant Sci. 2018 Oct 19;9:1503. doi: 10.3389/fpls.2018.01503 (PMC6202938; doi:10.3389/fpls.2018.01503)
Supplement: Supplementary file 1 [file Data_Sheet_1.docx]

**KEY RESOURCES TABLE**

| REAGENT or RESOURCE | SOURCE | IDENTIFIER |
| --- | --- | --- |
| Antibodies | | |
| anti-H3K4me2 | MILLIPORE | Cat#07-030 |
| anti-H3K4me3 | MILLIPORE | Cat#07-473 |
| anti-H3K27me2 | MILLIPORE | Cat#07-452 |
| anti-H3K9ac | MILLIPORE | Cat#06-866 |
| anti-H4ac | MILLIPORE | Cat#07-352 |
| anti-RNAPII | ABCAM | Cat#ab817 |
| anti-WHIRLY1 | This paper | N/A |
| anti-GFP | TRANSGEN BIOTECH | Cat#HT801-01 |
| anti HA-Tag Mouse Monoclonal Antibody | CWBIOTECH | Cat#CW0092M |
| anti-H3 | AGRISERA | Cat#AS10710 |
| Goat Anti-Rabbit IgG | CWBIOTECH | Cat#CW0103M |
| Goat Anti- Mouse IgG | CWBIOTECH | Cat#CW0102M |
| Chemicals, Peptides, and Recombinant Proteins | | |
| Ampicillin | AMRESCO | Cat#0339 |
| Kanamycin | AMRESCO | Cat#0408 |
| 3-Deazaneplanocin A（DZnep） | CAYMAN | Cat#13828 |
| Formaldehyde solution | SIGMA-Aldrich | Cat# 252549 |
| Complete Protease inhibitor tablet | ROCHE | Cat#4693116001 |
| Sarkosyl | SIGMA | Cat#L7414 |
| B2 RNA | Espinoza et al., 2004 | N/A |
| Critical Commercial Assays | | |
| Protein A agarose/Salmon Sperm DNA | MILLIPORE | Cat#16-157 |
| TransZol UP reagent | TRANSGEN BIOTECH | Cat#ET111-01 |
| DNaseI | THERMO SCIENTIFIC | Cat#EN0521 |
| RevertAid First Strand cDNA Synthesis Kit | THERMO SCIENTIFIC | Cat#K1622 |
| DNA Ligation Kit verb 2.1 | TAKARA | Cat#6022 |
| Active & Motif Chromatin Assembly Kit | ACTIVEMOTIF | Cat#53500 |
| Universal DNA purification kit | TIANGEN | Cat#DP214-03 |
| HeLa Core Histones | ACTIVEMOTIF | Cat#53501 |
| KOD SYBR qPCR Mix | TOYOBO | Cat#QKD-201 |
| PrimerSTAR max Premix | TAKARA | Cat#R045A |
| 2×UltraSYBR Mixture | CWBIOTECH | Cat#cw0956c |
| SDS-PAGE Gel Kit | CWBIOTECH | Cat#CW0022 |
| Deposited Data | | |
| Microarray data | Lin et al., 2018 | N/A |
| Experimental Models: Organisms/Strains | | |
| T­DNA mutant line of A. thaliana (insertion in  At1g14410) | European Arabidopsis Stock Centre | Salk_023713 |
| WHIRLY1 complementary line (PWHY1) | Miao et al., 2013 | N/A |
| WHIRLY1 overexpression line (*oepnWHY1*) | Miao et al., 2013 | N/A |
| nWHIRLY1 overexpression line (oenWHY1) | Miao et al., 2013 | N/A |
| T­DNA mutant line of A. thaliana (insertion in  AT3G18520) | Liu et al., 2013 | SALK_004027 |
| ATX1 knockout line | Ding et al., 2012 | N/A |
| ATX1 overexpression line | Ding et al., 2012 | N/A |
| Software and Algorithms | | |
| GraphPad Prism 6.0 | GraphPad | https://www.graphpad.com/scientific-software/prism/ |
| Heml 1.0 | The cuckoo workgroup | http://hemi.biocuckoo.org/ |
| Other | | |
| See Table S1-3 for primer sequences | This paper | N/A |

Contact for Reagent and Resource Sharing

Further information and requests for reagents may be directed to, and will be fulfilled by the corresponding authors Ying Miao (ymiao@fafu.edu.cn).

**Experimental Model and Subject Details**

*Arabidopsis thaliana*

All the plants used were *Arabidopsis thaliana (L.) Heynold ecotype Columbia 0* background, WHIRLY1 T-DNA insertion line *Salk_023713* (*why1*) were provided by the European Arabidopsis Stock Centre, while WHIRLY1 overexpression mutants (*oepnwhy1* and *oenwhy1*), WHIRLY1 complementary line (PWHY1) which harbors its own promoter and WHIRLY1 cds plus HA target were constructed at previous research ([Miao et al., 2013](#_ENREF_57)). Seeds were germinated on wet filter paper after 48h of vernalization. Then they were then transplanted in pots in vermiculite in a climatic chamber with a 13-h-light (100 μE/h) /11-h-dark photoperiod, 22°C/18°C day-night temperature regime, and 60% relative humidity. Rosette leaves were labeled with different colored threads after emergence ([Hinderhofer and Zentgraf, 2001](#_ENREF_31)). For Dznep treatment, 5^th^-8^th^ rosette leaves from PWHY1 mutants at 6^th^ week were exposed to 0,1,5, 10μM Dznep dissolved in DMSO.

**Method Details**

Measurements of Chlorophyll Fluorescence and Chlorophyll Content

Chlorophyll fluorescence of leaf 5 from different developmental plants were measured using a Pocket PEA Chlorophyll Fluorimeter (Hansatech) after 15-minimum dark incubation. The average Fv/Fm value of leaf 5 from at least 12 individual plants was calculated. Chlorophyll concentrations of leaf 5 from 12 different developmental plants were measured by Dualex 4. Three points（top, left, right） of each leaf were detected.

mRNA Preparation and qRT-PCR Analysis

Total RNA from 5^th^ to 8^th^ rosette leaves was isolated according to the manufacturer’s protocol of TransZol UP (TRANSGEN) and was then treated with RNase-free DNaseI (EN0521, Thermo Scientific). First-strand cDNA was generated from 1μg portion of total RNA using RevertAid First Strand cDNA Synthesis Kit (K1622, Thermo fisher), following the instruction. First strand cDNA was diluted 3 times. PCR was performed to analyze the expression of genes in the CFX96 (Bio-Rad) in a whole volume of 15 μl, including 2μl of 2×UltraSYBR Mixture (cw0956c, CWBIOTECH), 0.5μl of each gene-specific primer(10μM) and 1μl cDNA. To determine the relative expression rate, data were normalized to the expression level of wild-type or of 5-week-old plants (which were set to 1) after normalized to the internal control of *GAPC2* (AT1G13440). Additionally, three technical replicates of three biological replicates and the determination of a melting curve of the amplified PCR products were carried out. The primer sequences used are described in Table S1.

In vivo ChIP Assay

The ChIP assay was performed with a modified method (Gendrel, 2005). About 0.75g leaves (5^th^ to 8^th^ leaves of mutants and wild-type plants during different developmental stages) were fixed with 1% formaldehyde for 15 min and quenched with 2.5ml 2M glycine. After grinding to a fine powder with a mortar and pestle in liquid nitrogen, nuclei in leaves were extracted, and the chromatin contained was resuspended in 1% SDS nuclear lysis buffer with the addition of complete protease inhibitor cocktail tablets (Roche Diagnostics). Then the extract sonicated to fragment the DNA to a size range from 200-1000 bp, with a peak of intensity of about 500 bp. After centrifugation, the supernatant was diluted by ChIP dilution buffer (1.1% TritonX-100, 1.2mM EDTA, 16.7mM Tris-HCl, pH8.0, 167mM NaCl) and then precleared with protein A–agarose beads. The precleared supernatants were immunoprecipitated overnight at 4°C with histone modification antibodies, or were processed with no antibody and added as a control (mock precipitation). The antibody protein complexes were isolated by binding to protein A-agarose beads then reverse the formaldehyde cross-linking after elution. The immunoprecipitated DNA was isolated by Universal DNA purification kit (TIANGEN, DP214-03). Purified DNA was analyzed by real-time PCR with specific primers (see Supplemental Table S2). Relative histone modification levels in WT during leaf development were therefore normalized to the input while the enrichment in WHIRLY1 mutants was normalized to WT again, referring to ΔΔCt method (https://www.qiagen.com/us/resources/resourcedetail?id=34e05db7-689c-4abc-bde6-6488d097394f&lang=en).

Chromatin assembly and transcription assays

Chromatin was assembled and transcription assays were performed as described by Active & Motif Chromatin Assembly Manuel (www.activemotif.com). pG5ML with promoter of *WRKY53* including mutated WRKY53II (mutant (GTNNNGGT) m1 or mutant (CTNNNNAAAT) m2 WHIRLY1 binding motif) ([Miao et al., 2013](#_ENREF_57)) or mutated TATA-box or wild-type fragment was used as DNA template. The mutant TATA was created using site-directed mutagenesis (Agilent Technologies). The nuclear extracts were depleted of TFIID essentially as described ([Baek et al., 2002](#_ENREF_5)) with a few modifications. Briefly, the extracts were purified over Protein A Sepharose CL4B (Pharmacia) crosslinked to TBP, WHIRLY1 antibodies with dimethyl pimelimidate (DMP) (Sigma). The depletion experiments were carried out in BC buffer (20 mM Tris pH 7.9, 20% glycerol, 0.1 mM EDTA) containing 500 mM KCl (BC500) and the depleted extracts were dialyzed in BC100 before freezing. Equivalent amounts of each TFIID complex, as determined by immunoblot analysis of TBP and WHY1, were examined in the in vitro run-on transcription assays performed with ΔIID NE.

*In vitro* ChIP

In vitro ChIP assays were performed as described ([Lauberth et al., 2013](#_ENREF_44)). In vitro ChIP assays were performed in the presence of recombinant and/or affinity purified basal transcription factors (TFIIA, TFIIB, TFIIE, TFIID, TFIIF, TFIIH, PC4, Mediator, and RNAPII). Briefly, assembled chromatin was digested by microccocal nuclease to generate mononucleosomes, then incubated with antibody at 4 ºC overnight. The immune complexes were incubated with Protein A or G beads (containing salmon sperm DNA) (Millipore) at 4 ºC for 1 hr then the bound DNA was eluted, purified, and analyzed by qRT-PCR. Results were quantified relative to inputs ([Lauberth et al., 2007](#_ENREF_43)) and the levels of H3K4me3, H3K9ac are given relative to the total H3 levels. In vitro ChIP to detected the effect of WHIRLY1 on transcription stage was performed in the presence or absence of 0.01% sarkosyl, which inhibits PIC assembly but does not affect elongation by pre-formed complexes ([Cai and Luse, 1987](#_ENREF_9); [Hawley and Roeder, 1987](#_ENREF_29)) or 800nM B2 RNA, which inhibits transcription prior to PIC formation([Espinoza et al., 2004](#_ENREF_22)).

Run-on transcript assay

Reference to method described by Pelechano et al., (2009). After chromatin assembly and in vitro ChIP, TFIID complexes are isolated and incubated with labeled nucleotides, and TBP gene in the process of being transcribed are detected by hybridization of extracted RNA to gene specific probe TBP on a blot (Khraiwesh, 2011). X-ray exposed the film.

Pelechano, V., Jimeno-González, S., Rodríguez-Gil, A., García-Martínez, J., Pérez-Ortín, J.E., Chávez, S (2009).  Regulon-specific control of transcription elongation across the yeast genome. *PLoS Genet*. **5** (8): e1000614.

Khraiwesh B (2011). Using nuclear run-on transcription assays in RNAi studies. Methods Mol Biol. 744:199-209. doi: 10.1007/978-1-61779-123-9_14.

**Quantification and Statistical Analysis**

Statistical significance of the data otherwise stated was assessed using Student’s t test which is represented by * at p < 0.05，** at p< 0.01, *** at p<0.001. In figure legends n means the number of independent experiments. Statistical analysis was performed using GraphPad Prism6. Graphs show mean ± SD.
