# Supplementary material for: WHIRLY1 Occupancy Affects Histone Lysine Modification and WRKY53 Transcription in Arabidopsis Developmental Manner
Source: Front Plant Sci. 2018 Oct 19;9:1503. doi: 10.3389/fpls.2018.01503 (PMC6202938; doi:10.3389/fpls.2018.01503)
Supplement: Supplementary file 2 [file Image_1.pdf]

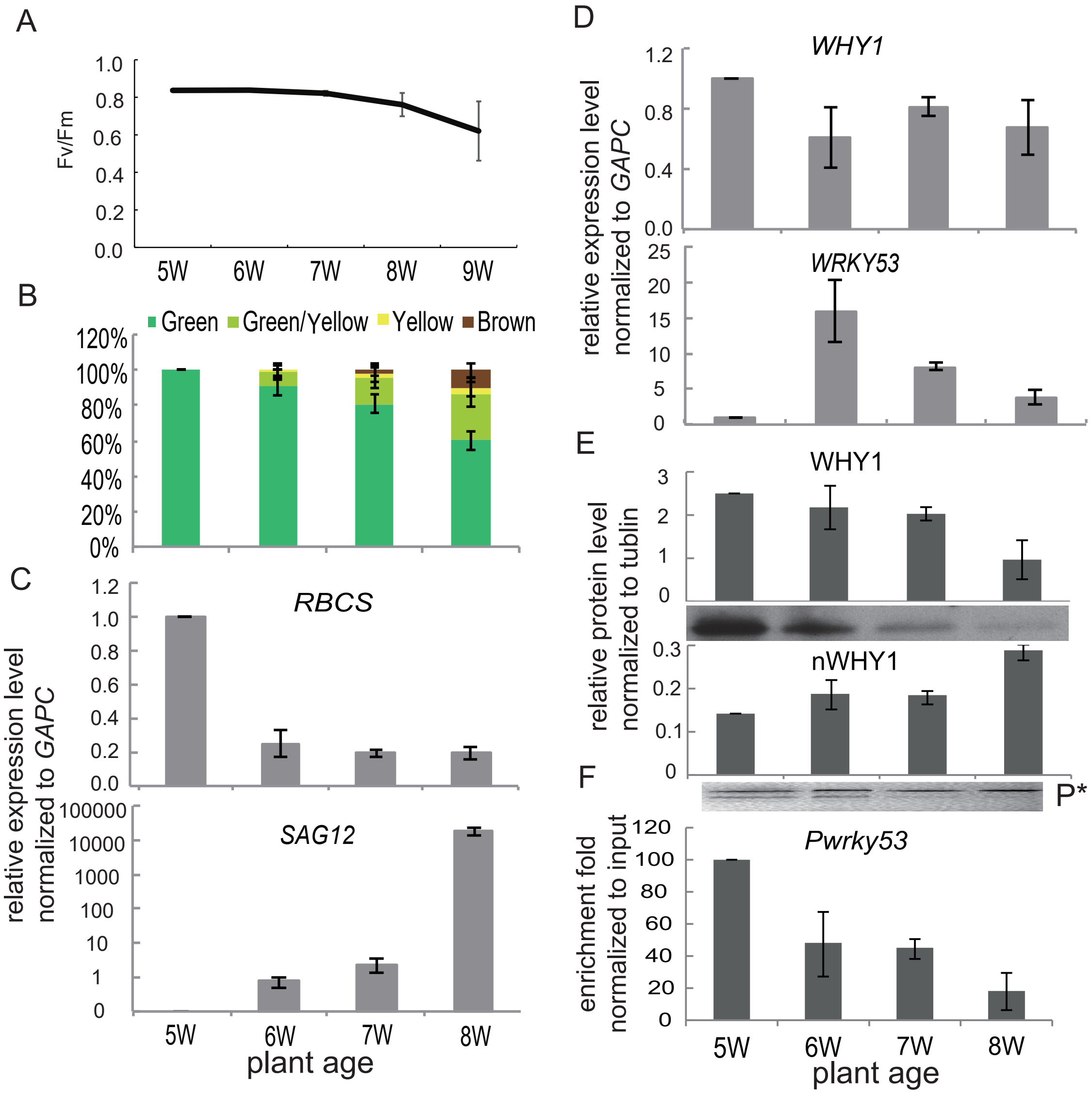

Supplemental Figure S1 WHIRLY1 binds on the promoter of *WRKY53* and represses its transcription at senescence initiation stage

A. Fv/Fm of leaf 7th and in wild-type plants from 5th week to 9th week.

B. Senescent leaf fraction from wild-type plants from 5th week to 9th week. Means and SD of at least 12 independent measurements are shown in (A) and (B). Error bars represent standard error.

C. Expression of senescence-associated genes in wild-type plants during leaf development from 5th week to 8th week by qRT-PCR.

D. Expression of *WHIRLY1* and *WRKY53* in wild-type plants during leaf development from 5th week to 8th week by qRT-PCR. The mRNA used in (C) and (D) was isolated from 5th to 8th leaves at different developmental stages. The transcript level in each case was normalized to that of *GAPC2* as a reference gene, and the expression level at 5W was set as 1. Three biological replicates and three technique replicates were used to analyze. Error bars represent standard error.

E. WHIRLY1 amount and status change during plant aging.

Total WHIRLY1 protein and nuclear WHIRLY1 protein (nWHY1) or nuclear WHIRLY1 treated with phosphatase isolated from 5th to 8th week Arabidopsis rosette leaves. Asterisk indicates the phosphorylated form of WHIRLY1. Western blot with antibody against tublin used as loading control.

F.The binding affinity of WHIRLY1 to GTCAAAT.AAAAT element at promoter of *WRKY53* (*Pwrky53*) by ChIP experiment.

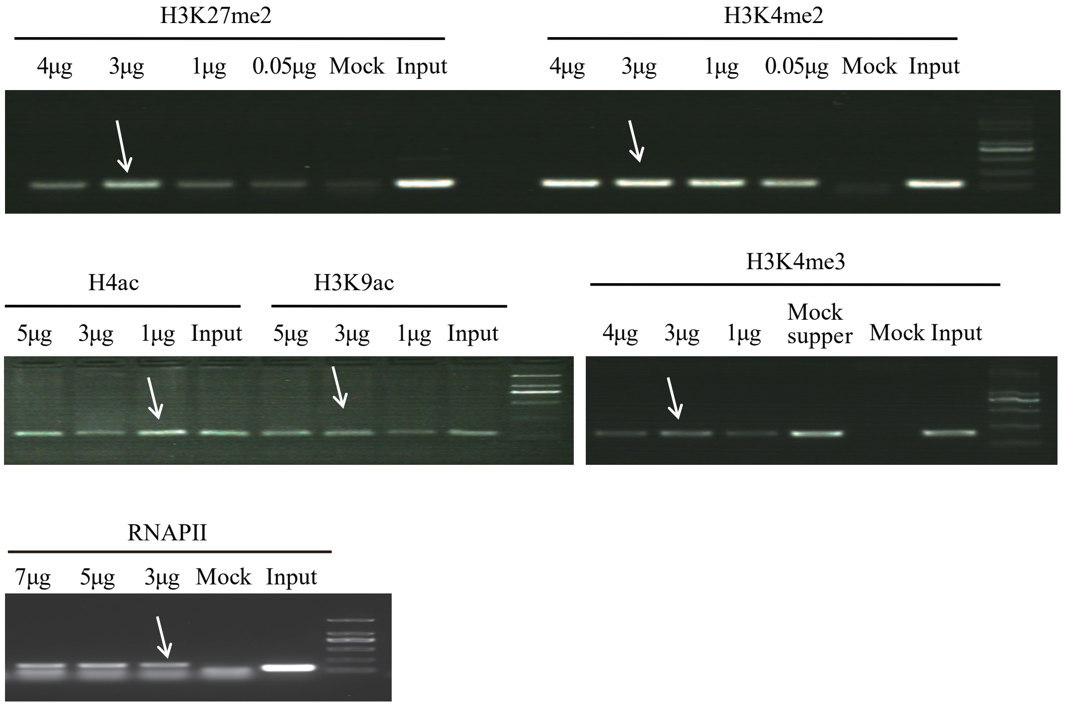

Supplemental Figure S2 Dosage of antibodies relative to a fixed amount of sample Chromatin was isolated from 1.5g leaves, sonicated and divided into five slices for four “IP” and “Mock” after 1:10 dilution. Different dosages of antibodies were used for immunoprecipitation. PCR were performed with ACT7, MULE or WRKY53P primers. The dosages of different antibodies used subsequently were signed with arrow. H3K4me2 (07-030), H3K4me3 (07-473), H3K27me2 (07-452), H4ac (06-866), H3K9ac (07-352) bought from Upstate Biotechnology Company, RNAPII, Abcam, company（ab817）.

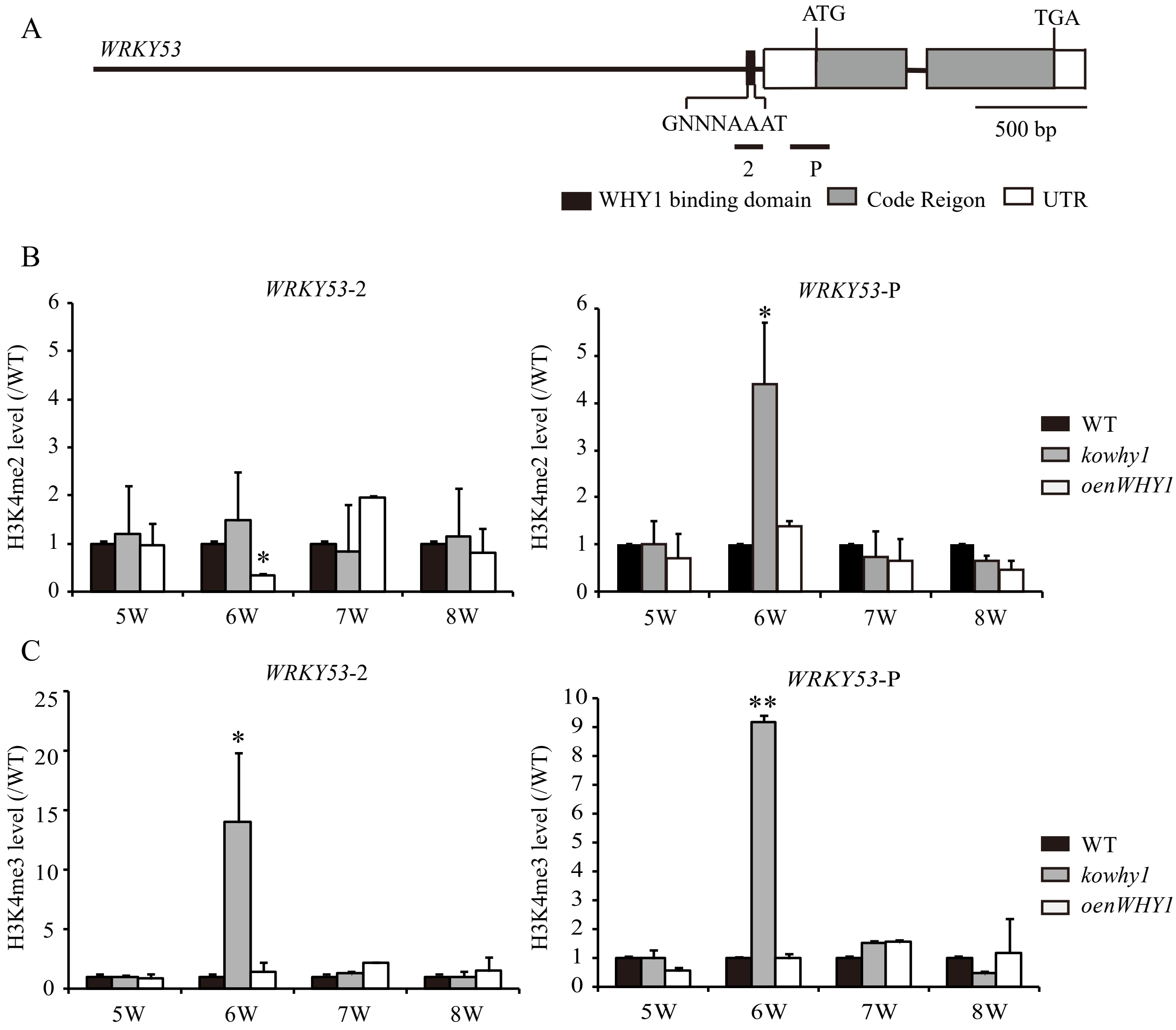

Supplemental Figure S3 H3K4me2 and H3K4me3 at WRKY53 in WHY1 mutants during plant aging from 5th week to 8th week analyzed by ChIP-qPCR.
A. Schematic diagram of the genomic structure of the WRKY53 gene. The thick lines with number represent qPCR amplicons in different regions of *WRKY53* gene.
B-C. ChIP analyses of changes in H3K4me2(B), H3K4me3(C) at promoter (site 2) and transcriptional (site P) regions of *WRKY53* in WHY1 mutants from 5W to 8W. The relative level was normalized to WT. Error bar show the standard deviation(n=3). Asterisk indicate significant differences (*P < 0.05 and **P<0.01) based on Student's t-test analyzed by Graphpad prism6 software.

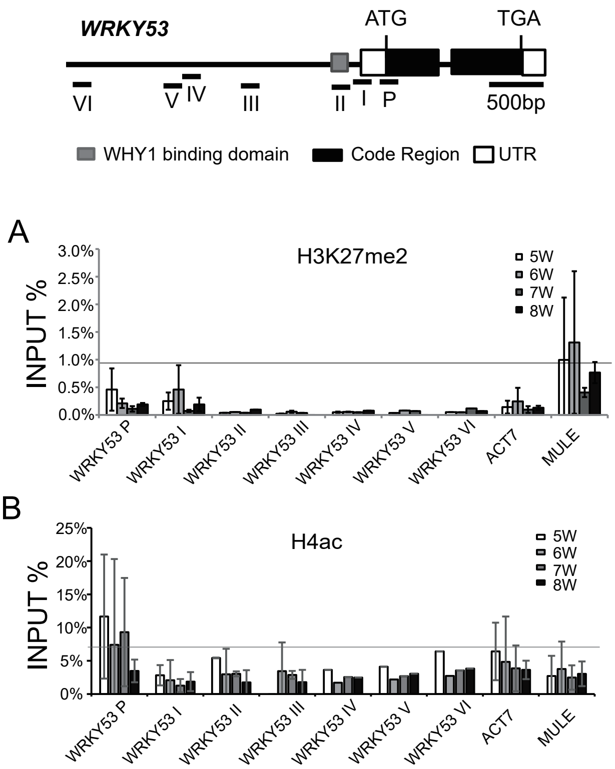

Supplemental Figure S4 Histone modification enrichment at the promoter regions and translation start region of *WRKY53*
Schematic diagram of the genomic structure of the *WRKY53* gene. The lines with number represent qPCR amplicons in different regions of *WRKY53* gene. Gary box, black box and blank box represent WHIRLY1 binding domain (gray), exon (black) and untranslated regions (blank). A.-B. ChIP analyses of changes in H3K27me2 (B), H4ac (C) levels occupancy at different regions of *WRKY53* from 5th to 8th week. The relative level was normalized to INPUT DNA. Error bar show the standard deviation (n=3). Asterisk indicate significant differences (*P < 0.05 and **P<0.01) based on Student's t-test.

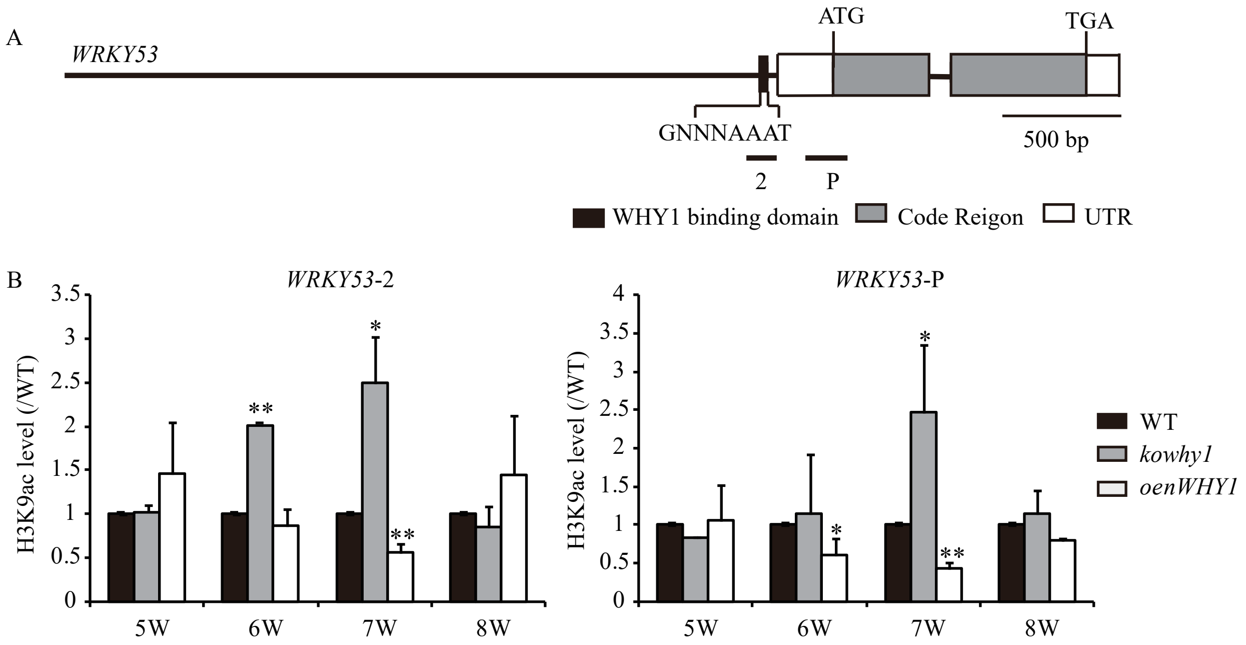

Supplemental Figure S5 H3K9ac at *WRKY53* in *WHY1* mutants during plant aging from 5th week to 8th week analyzed by ChIP-qPCR.

A. Schematic diagram of the genomic structure of the *WRKY53* gene. The thick lines represent qPCR amplicons in different regions of *WRKY53* gene.

B-C. ChIP analyses of changes in H3K9ac (B) at promoter (site 2) and transcriptional (site P) regions of *WRKY53* in WHY1 mutants from 5W to 8W. The relative level was normalized to WT. Error bar show the standard deviation(n=3). Asterisk indicate significant differences (*P < 0.05 and **P<0.01) based on Student's t-test analyzed by Graphpad prism6 software.

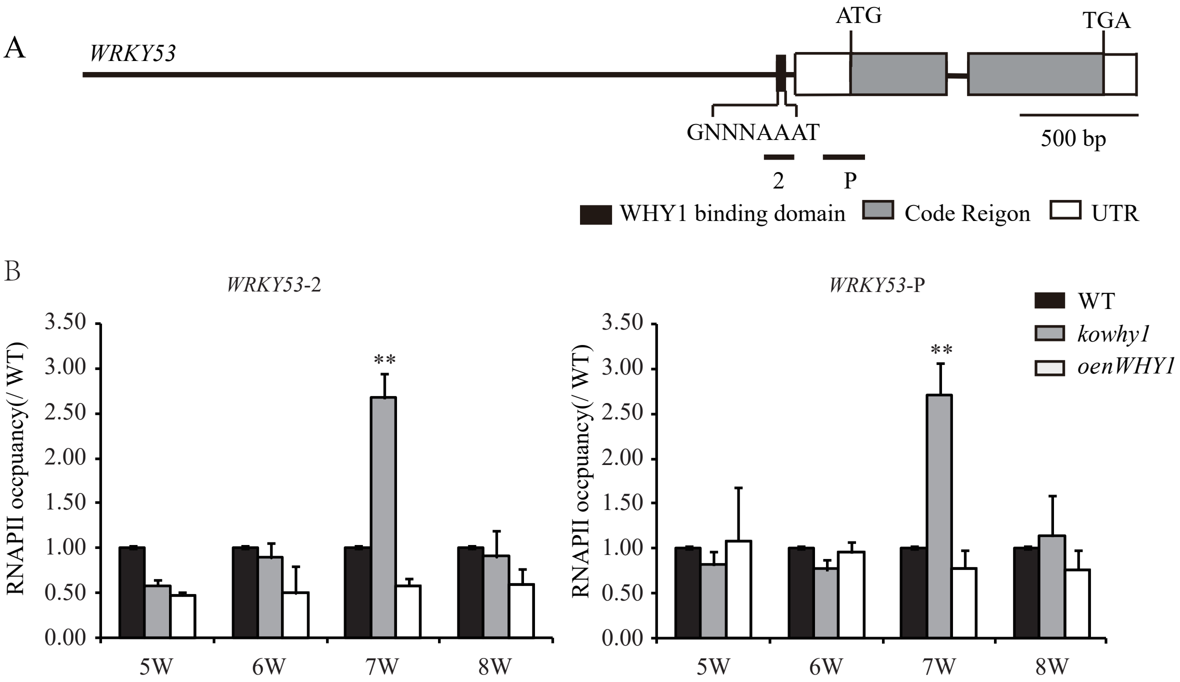

Supplemental Figure S6 RNAPII occupancy at *WRKY53* in *WHY1* mutants from 5th week to 8th week analyzed by ChIP-qPCR.

A. Schematic diagram of the genomic structure of the *WRKY53* gene. The thick lines with number represent qPCR amplicons in different regions of *WRKY53* gene.

B. ChIP analyses of changes in RNAPII occupancy at promoter (site 2) and transcriptional (site P) regions of *WRKY53* in *WHY1* mutants from 5W to 8W. The relative level was normalized to WT. Error bar show the standard deviation(n=3). Asterisk indicate significant differences (*P < 0.05 and **P<0.01) based on Student's t-test analyzed by Graphpad prism6 software.

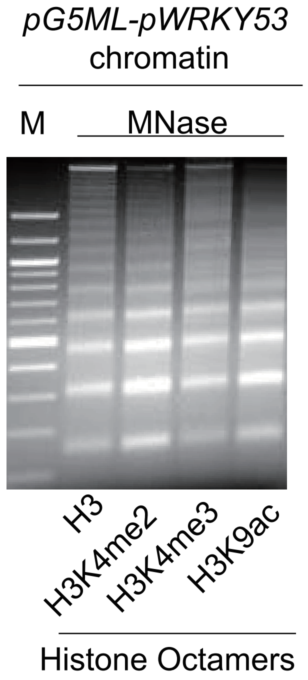

Supplemental Figure S7 MNase digestion showing similar nucleosome spacing for chromatin assembled with unmodified H3 and H3K4me2/3 or H3K9ac-modified octamers.

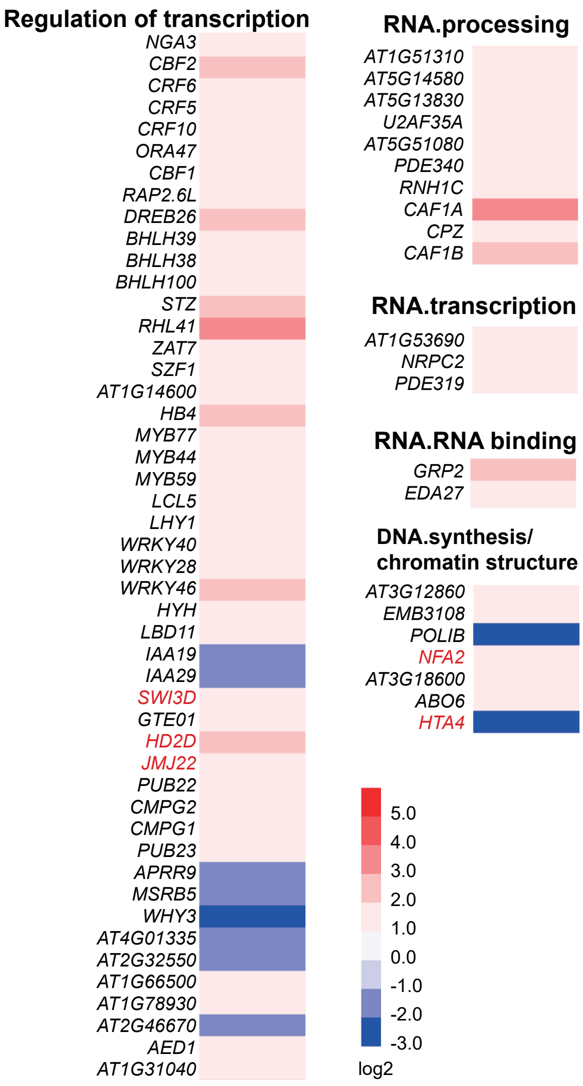

Supplemental Figure S8 WHIRLY1 affects the expression of histone modification related genes

Heat map of difference expression genes in *why1* mutant compared with wild-type plants selected from microarray data.
