# Supplementary material for: WHIRLY1 Occupancy Affects Histone Lysine Modification and WRKY53 Transcription in Arabidopsis Developmental Manner
Source: Front Plant Sci. 2018 Oct 19;9:1503. doi: 10.3389/fpls.2018.01503 (PMC6202938; doi:10.3389/fpls.2018.01503)
Supplement: Supplementary file 3 [file Data_Sheet_2.PDF]

Table S1 Primers used for ChIP-qPCR

TAIR number	Primer Name	Sequences (5' to 3')
AT4G23810	WRKY53-1-FP	TCAAACACTGAAAATCCAATGCCT
	WRKY53-1-RP	AATGAGAGTGTTATAGGATTGGTGA
	WRKY53-2-FP	CTCTGGCCCTATACTTCCTTATAC
	WRKY53-2-RP	GGTTTGGTATATAAAGGCATTGGAT
	WRKY53-3-FP	CCCATGAGTGAGTTTTGACCC
	WRKY53-3-RP	GCTGAAGTTGACACACACGAG
	WRKY53-4-FP	CGCAAAAGAGAGTGACGCC
	WRKY53-4-RP	TTTCAAACCTCTCTCCGCGT
	WRKY53-5-FP	TAGGGTGCAAAGTGCCAAGAT
	WRKY53-5-RP	AATGGCGTCACTCTCTTTTGC
	WRKY53-6-FP	ACCGGCTCTAAAGACAGTTGG
	WRKY53-6-RP	AAGACGTGCTGTCCTCTGAA
	WRKY53-P-FP	GATCATATCATTCTTTCAGTCCATC
	WRKY53-P-RP	CCAACTTAACATATCTCTTCCTTCC
AT2G15810	MULE-ChIP-FP	GCTCCCTCTATGGTTTACCCTCTACTG
	MULE-ChIP-RP	GTTTCCGTTGCTTGTGGCTTCTATG
AT5G09810	ACT7-ChIP-FP	CGTTTCGCTTTCCTTAGTGTTAGCT
	ACT7-ChIP-RP	AGCGAACGGATCTAGAGACTCACCTTG

Table S2 Primers used for qPCR

TAIR number	Gene Name	Primer sequences（5' to 3'）
AT5G26040	*HDA2*	FP：ACCCATTCCGAAAGCAGGTTGG
		RP：ATCGCCCATCCACGTTCTGTTG
AT5G61060	*HDA5*	FP：AGGAGGAGTTGCTTTCACCAACC
		RP:TGAACTGCTCAAGCGTGATACGG
AT5G63110	*HDA6*	FP:AACCTCGCATCTGGAGTGGAAC
		RP:ATCTTCACCGGTAGAGTCCCTGTC
AT1G08460	*HDA8*	FP:AGCTGGTGGTTCCTGCAGTTAG
		RP:AAAGCGCTAGAGTCTTGACCAAC
AT3G18520	*HDA15*	FP:GATTTGATGCGGCTAGAGGAGACC
		RP:AATAGCCAGCCGGAGTCACATC
AT2G31650	*ATX1*	FP:ACCAACAGAACATGTAGACTTCCG
		RP:AGAGTCTGTCACAACCTTTCCAAG
AT1G05830	*ATX2*	FP:ACTTGTCGAGTGGCATATCATCCG
		RP:GCCTATCCTCATCTGCAAGCTC
AT2G27840	*HD2D*	FP:AGCAGCCACATCTTCCTCTTTCAC
		RP:TCCCTGGCTTAATCTCGATACCC
AT2G19480	*NAP1*	FP:AGTCATCAGCTGGGCACAAGAAG
		RP:TTACATTCCGGTGGCCTCTCAC
AT4G34430	*CHB3*	FP:TGGTGCCTAATCATTGCGGTTGG
		RP:GGCAATGAACGTTCCTCAAGCG

AT4G13570	*HTA4*	FP:TTCTGCACATAGTAGTGTTGGT<br>RP:CCAACTGAAGAACCTCTGTAGT
AT5G06550	*JMJ22*	FP:ATTGAGTGTATATGCAAAGCCG<br>RP:TCGATTCCTCCAGGTTAATCAC
AT5G45890	*SAG12*	FP:GCTTTGCCGGTTTCTGTTG<br>RP:GTTTCCCTTTCTTTATTTGTGTTG
AT1G66580	*SAG24*	FP:CTAAGTTTAAGTTCCCTGGTCGTC<br>RP:CACAATCCTCTTCATCGCTCTC
At5g14930	*SAG101*	FP:CACCGATCTGCAGAAGTAGTAGC<br>RP:GAGCAGAGGAATGGGAAAGG
AT5G13180	*ANAC083*	FP:CAGACCAAACAGATGATAAAACAAAAC<br>RP:GAAGCATGATCGGAAGAAGGAG
At4g23810	*WRKY53*	FP:CAGACGGGGATGCTACGG<br>RP:GGCGAGGCTAATGGTGGTG
AT5G10020	*SIRK*	FP:GTTTATAGTCTTGGGGTTGTTCTTC<br>RP:GATCACTTATATGCACCTTCTCTG
AT1G79850	*RPS17*	FP:CGAAGCCGGAGGAGATGAAC<br>RP:CGGGATACACAACAAACAAACAGAAG
AT1G67090	*RBCS*	FP:CATCACAAGCAATGGGGGAAGAG<br>RP:CCATTTGTTGCGGAGAAGGTAGTC
AT3G54890	*LHCA1*	FP:GCCGGGAATGTTGGTCGTATCAG<br>RP:CAAACCCAAAGTCACCAGGAGCAG
AT1G13440	*GAPC2*	FP:ACCACTGTCCACTCTATCACTGC<br>RP:TGAGGGATGGCAACACTTTCCC

Table S3 Primers used for vector construction

Primer name	Sequences (5' to 3')
ATX1-Y2H-AD/BD_FP	TCCACCCGGGGGCCATGGCAATGGCGTGTTTTTCTAACGAAACCCAGAT
ATX1-Y2H-AD/BD_RP	GCTCGAGCTCGTCGACTTCTGCGGTCCAGTCTATTAGATCACAA
ATX2-Y2H-AD/BD_FP	TCCACCCGGGCATGATTTCAATGTCGTGTGTCCCAAAAGAAGA
ATX2-Y2H-AD/BD_RP	GCAGCTCGAGGTCGACGGACTCTGTCCACTCTTTTAACTCACAGCGA
AtSUVH2-Y2H-AD/BD_FP	TACGGGATCCCCATGGCGATGAGTACATTGTTACCATTTCCT
AtSUVH2-Y2H-AD/BD_RP	GCAGCTCGAGGTCGACGTTGCAGATGGCGAGCTTGC
HDA15-Y2H-AD/BD_FP	GGCCGAATTCATGGTTGTAGAAACTATCGAGAGGTC
HDA15-Y2H-AD/BD_RP	GCCGCTGCAGGAGCTCCGACGGATTAGGAAGAATGCTTTC
HDA19-Y2H-AD/BD_FP	AATTCCCGGGAATGGATACTGGCGGCAATTCGCTGGC
HDA19-Y2H-AD/BD_RP	GCCGCTGCAGCTCGAGTGTTTTAGGAGGAAACGCCTGCTCCGC-
HDA6-Y2H-AD/BD_FP	GGCCGAATTCATGGAGGCAGACGAAAGCGGCATC
HDA6-Y2H-AD/BD_RP	TTATGCGGCCGCGAGCTCAGACGATGGAGGATTCACGTCTGGCTC
WDR5a-Y2H-AD/BD_FP	TCCACCCGGGCATGGCAGAGGAAATTCCAGCAACAGC
WDR5a-Y2H-AD/BD_RP	GCAGCTCGAGCTGCAGTTCTTTCTTCTGTGTCCAAATCCTTACTGTCTTGTC
Ash2-Y2H-AD/BD_FP	ATGGCCATGGGAATTCATGGAGTCTCTTCAATCAAATTCC
Ash2-Y2H-AD/BD_RP	TTATGCGGCCGCGAGCTCACTCTTCATATCCTCAGAACCATTAG
